# Experience of being a worker with a disability in the Colombian formal sector

**DOI:** 10.15649/cuidarte.4648

**Published:** 2025-12-17

**Authors:** Luz América Martínez-Álvarez, Stephania Hermann-Agudelo, Cecilia Andrea Ordóñez-Hernández

**Affiliations:** 1 Institución Universitaria Antonio José Camacho, Cali, Colombia. E-mail: lamericamartinez@admon.uniajc.edu.co Institución Universitaria Antonio José Camacho Cali Colombia lamericamartinez@admon.uniajc.edu.co; 2 Universidad de San Buenaventura, Cali, Colombia. E-mail: shermanna@usbcali.edu.co Universidad de San Buenaventura Cali Colombia shermanna@usbcali.edu.co; 3 Universidad del Valle, Cali, Colombia. E-mail: cecilia.ordonez@correounivalle.edu.co Universidad del Valle Cali Colombia cecilia.ordonez@correounivalle.edu.co

**Keywords:** People with Disabilities, Surveillance of Workers Health, Occupational Health, Job Description, Social Adjustment, Personas con Discapacidad, Vigilancia de la Salud del Trabajador, Salud Laboral, Perfil Laboral, Ajuste Social, Pessoas com Deficiência, Vigilância em Saúde do Trabalhador, Saúde Ocupacional, Descrição de Cargo, Ajustamento Social

## Abstract

**Introduction:**

In Colombia, 3 out of 10 WWDs have a formal employment contract. When workers are the ones required to adapt to the company, rather than the company adapting to them, barriers persist, limiting genuine inclusion.

**Objective:**

To understand the experiences of WWDs employed in the formal sector of the economy through an employment contract.

**Materials and Methods:**

A qualitative study with a phenomenological approach was conducted. Three men and two women participated: a man with a visual impairment and four individuals with motor disabilities, all formally employed in Cali, Colombia, and selected through purposive sampling. Data were collected through in-depth interviews, and the phenomenological analysis followed three phases: description, reduction, and interpretation. The lifeworld existentials of lived body, lived space, lived time, and lived human relations, as well as perceptions of working conditions, were categorized.

**Results:**

Workers reported access limitations and restrictions in work tasks due to their disabilities. Despite receiving support from their companies, a lack of commitment to adapting spaces and processes for inclusion was observed.

**Discussion:**

WWDs perceive reasonable accommodations as a desirable factor to facilitate adaptation. However, companies do not consistently implement such adaptations.

**Conclusion:**

WWDs develop adaptation strategies, making a personal commitment to integrate into the workplace, often without specific accomodations from the company. Once integrated, they perform autonomously and productively, under conditions similar to those of their colleagues without disabilities.

## Resumen

**Introducción:**

En Colombia, 3 de cada 10 trabajadores con discapacidad cuentan con contrato laboral. Cuando el trabajador debe ajustarse a la empresa y no al revés, las barreras no desparecen, y limitan la inclusión real.

**Objetivo:**

Comprender las experiencias de trabajadores con discapacidad vinculados al sector formal de la economía mediante contrato laboral.

**Materiales y métodos:**

Se realizó un estudio cualitativo con enfoque fenomenológico. Participaron 3 hombres y 2 mujeres: un hombre con discapacidad visual y 4 personas con discapacidad motora, todos con vinculación laboral formal en Cali, Colombia, seleccionados mediante muestreo propositivo. La información se recolectó mediante entrevistas en profundidad, y el análisis fenomenológico se realizó en tres fases: descripción, reducción e interpretación. Se categorizaron los existenciales cuerpo, espacio, tiempo y relaciones humanas, junto con la percepción de las condiciones laborales.

**Resultados:**

Los trabajadores señalaron limitaciones de acceso y restricciones en tareas laborales debido a la discapacidad. A pesar de recibir apoyo de la empresa, se observó una falta de compromiso en adaptar los espacios y procesos para la inclusión.

**Discusión:**

Los resultados trabajadores con discapacidad perciben los ajustes razonables como un factor deseable para facilitar su adaptación. Sin embargo, las empresas no implementan dichas adaptaciones de forma constante.

**Conclusiones:**

Los trabajadores con discapacidad desarrollan estrategias de adaptación, asumiendo un compromiso personal para integrarse en el entorno laboral, a menudo sin ajustes específicos de la empresa. Una vez integrados, se desempeñan de manera autónoma y productiva, en condiciones similares a las de sus compañeros sin discapacidad.

## Resumo

**Introdução:**

Na Colômbia, 3 em cada 10 trabalhadores com deficiência possuem contrato de trabalho. Quando os trabalhadores precisam se adaptar à empresa e não o contrário, essas barreiras não desaparecem e limitam a inclusão real.

**Objetivo:**

Compreender as experiências de trabalhadores com deficiência vinculados ao setor formal da economia por meio de um contrato de trabalho.

**Materiais e métodos:**

Foi realizado um estudo qualitativo com abordagem fenomenológica. Participaram três homens e duas mulheres: um homem com deficiência visual e quatro pessoas com deficiência motora, todos com contrato de trabalho formal em Cali, Colômbia, selecionados por amostragem intencional. As informações foram coletadas por meio de entrevistas em profundidade, e a análise fenomenológica foi realizada em três fases: descrição, redução e interpretação. Aspectos existenciais de corpo, espaço, tempo e relações humanas foram categorizados, juntamente com percepções sobre as condições de trabalho.

**Resultados:**

Os trabalhadores relataram limitações de acesso e restrições nas tarefas de trabalho devido à sua deficiência. Apesar de receberem apoio da empresa, observou-se falta de compromisso com a adaptação de espaços e processos para a inclusão.

**Discussão:**

Os resultados mostram que os trabalhadores com deficiência percebem adaptações razoáveis como um fator desejável para facilitar sua adaptação. No entanto, as empresas não as implementam consistentemente.

**Conclusões:**

Trabalhadores com deficiência desenvolvem estratégias de adaptação, assumindo um compromisso pessoal de integração ao ambiente de trabalho, muitas vezes sem ajustes específicos da empresa. Uma vez integrados, eles trabalham de forma autônoma e produtiva, em condições semelhantes às de seus colegas sem deficiência.

## Introduction

Persons with disabilities (PWDs) face significant disadvantages in the workplace, which translates into more limited access to employment opportunities^1^. Statistically, the average employment rate among this population is 44%, approximately half that of the 75% recorded for non-disabled individuals^2^. Furthermore, the inactivity rate among PWDs is 2.5 times higher, reaching 49%, compared with 20% for non-disabled people^3^. Prolonged unemployment compels many PWDs to accept jobs in the informal sector or engage in self-employment, both contexts characterized by the absence of social security benefits and labor protection^4^.

There are myths surrounding the hiring of PWDs associated with concerns that fragile health may be costly in terms of both medical treatments and workdays lost due to sick leaves^5^. It is presumed that, once hired, workers with disabilities (WWDs) cannot be dismissed because they are under special protection of the State. It is also believed that WWDs are less productive^6^, dependent, and require constant assistance^7^. There is a lack of knowledge about whether special hiring conditions apply to PWDs, and interacting with them is difficult because many people are unsure how to address differences in others^8^.

Previous research evidence has shown that WWDs develop diverse coping strategies in their work environments. Among these strategies, “body crafting” stands out, a process by which individuals manage their physical and health-related needs to balance work and personal life effectively. This strategy requires continuous adjustment according to their abilities and work demands^9^. Resilience also emerges as a fundamental capacity, encompassing emotional regulation and optimism, and supported by internal factors, such as acceptance of one's condition and external factors, such as social support^10^. Additionally, these individuals engage in “resourcing work,” which involves identifying and utilizing available resources to facilitate labor participation, as well as “restorative entrepreneurship,” which enables them to rebuild their identities and reclaim autonomy in the face of ableist marginalization^11^. The concept of the “adapted self” highlights how WWDs adapt to both physical and psychological environments, negotiating altered physical and sensory conditions while adapting to psycho-emotional challenges within a normative society^12^. These adaptations take the form of various coping strategies, including adapting to existing conditions, avoiding certain tasks or environments, and directly confronting barriers, reflecting different forms of agency in the face of structural constraints^13^.

Within this context, Schutz's social perspective provides a relevant theoretical approach to understand the work experiences of PWDs^14^. Schutz posits that individuals attribute meaning to their everyday experiences through what he calls “in-order-to motives” and “because motives.” The latter refers to previous experiences that condition and structure an individual’s current behavior, whereas “in- order-to motives” denote future expectations and goals that drive their actions^15^. For WWDs, previous experiences of exclusion may shape their“because motives,” influencing how they perceive and adapt to their workplaces. Conversely, the pursuit of independence, social inclusion, and economic stability may constitute their “in-order-to motives” for participating in work.

Schutz also underscores the importance of intersubjectivity, understood as the set of lived experiences through which individuals construct a shared world in interaction with others^15^. In the workplace, intersubjectivity manifests in the relationships among WWDs, their colleagues, supervisors, and the broader organization. Thus, their experiences reflect not only their individual adaptation efforts but also how meanings and attitudes toward disability are collectively constructed in the workplace^16^.

Despite policy advances in labor inclusion, various studies have examined the working conditions of WWDs and have pointed out that they still face challenges. Although WWDs attain a certain degree of job stability, notable disparities persist, particularly regarding gender, as men with disabilities tend to hold more stable positions than women^17^. This phenomenon indicates enduring gender biases in access to quality employment, limiting professional development and economic security of women with disabilities while heightening their vulnerability in the labor market.

Moreover, workplace mistreatment and discrimination remain common experiences for WWDs^18^. Such experiences affect their emotional well-being and job satisfaction due to persistent exclusionary attitudes and microaggressions that perpetuate stigma toward disability. Although some coworkers support the inclusion of PWDs, employers and supervisors tend to be more reluctant, especially in the case of people with intellectual disabilities, due to stereotypes that wrongly associate^19^ these conditions with low productivity or an increased need for supervision. These prejudices not only constrain formal employment opportunities for people with intellectual disabilities but also undermine cohesion and perceptions of fairness within organizations. This study is important given the need to demystify the formal employment of WWDs by examining successful cases of labor inclusion among PWDs^20^,^21^. The guiding research question was: What is the essence of the work experience of WWDs who hold formal employment contracts in the Colombian formal sector?

The objective of this study was to understand the essence of the work experience of WWDs who hold employment contracts in the Colombian formal sector.

## Materials and Methods

This study employed a qualitative case study design with a phenomenological approach grounded in Alfred Schutz's social perspective, which emphasizes understanding individuals’ actions in the social world through intersubjectivity and everyday experiences^14^,^15^. Within a constructivist paradigm, this approach enabled the analysis of how WWDs interpret and make sense of their work experiences, considering their interactions and shared expectations within professional settings. Consequently, this research constitutes a phenomenological case study^22^,^23^ based on statements^24^ of WWDs formally employed in companies located in the department of Valle del Cauca, Colombia.

Phenomenology and case study methodologies converge when the research aims to understand the lived experiences of individuals or groups in a specific context. The case study approach enables grasping the complexity of a concrete situation in which workers with a disability attribute meaning to their work experience within a particular institutional and social environment. This approach provides the narrative and contextual framework necessary to observe how the structures of the life- world (Lebenswelt) are actualized in practice.

Phenomenology, in turn, enables suspension of preconceived judgments and exploration of experiences as lived by the individuals. This ensures that the analysis is not limited to describing objective working conditions or technical adaptations but instead delves into how the individual experiences, interprets, and adapts to the work environment on a daily basis.

A total of five participants, three men and two women, were selected through purposive sampling^25^. The informants were contacted within the framework of a larger research project entitled “Systematization of labor inclusion practices for persons with disabilities,” conducted in service and manufacturing companies in Cali between 2020 and 2022. To recruit participants, a systematic process was undertaken to approach the business sector in Cali, Colombia. This process involved visiting 227 companies from various economic sectors during the second half of 2023 and was conducted in three phases: first, initial contact was made with human resources departments via email and telephone; second, on-site visits were made to companies that confirmed employing PWDs, during which potential participants were identified; and third, informational meetings were scheduled with potential participants. The inclusion criteria established were: having a certified disability, holding a formal employment contract lasting more than six months, and working in a company belonging to the formal sector of the economy. From this process, five WWDs met the criteria and expressed interest in participating: three men and two women, one with a visual impairment and four with motor disabilities.

Informants also needed to be articulate and willing to share their experiences. In accordance with ethical research standards, all participants signed informed consent forms, which ensured confidentiality and the voluntary nature of participation. In-depth interviews were conducted by an interdisciplinary research team composed of specialists in workplace inclusion and experts in qualitative methodologies. Each interview lasted approximately 90 minutes and was held during working hours in spaces provided by the companies, with prior authorization from the participants’ immediate supervisors, thereby facilitating participation without affecting work responsibilities.

The sample size was determined according to the criterion of theoretical saturation, which was reached when no new relevant information emerged from participants’ accounts and the analytical categories were sufficiently explored and substantiated.

Data were collected through in-depth, semi-structured interviews. A recruitment protocol was developed to describe the step-by-step interview process in detail to reduce procedural bias. An interview guide was also designed, which included the four phenomenological lifeworld existentials as main themes: lived body, lived space, lived time, and lived human relations, as well as working conditions.

Emergent categories related to worker adaptations, job characteristics, disability consequences in the workplace, and worker aspects were linked to the corresponding phenomenological main themes. (Table 1).

**Table 1:** Linking phenomenological main themes with emergent categories

Phenomenological main themes	Emergent categories	Linking
Lived body	Worker adaptations; Disability consequences	The body affected by a disability is experienced through work activities, and how workers with disabilities adapt physically and emotionally to tasks.
Lived time	Worker adaptations; Disability consequences	Workers with disabilities experience a change in the perception of working time (pace, breaks, agility, speed) and how this affects their productivity.
Lived space	Worker adaptations; Job characteristics	Physical work environment either promotes or limits mobility, accessibility, and functional performance for workers.
Lived human relations	Worker concept; Worker adaptations; Disability consequences	Workers with disabilities strive to maintain good relationships with colleagues and bosses and achieve the best possible productivity despite their difficulties. They are aware of the distinct social perceptions directed toward workers with disabilities.

The interviews were conducted in person at times and locations scheduled by the participants according to their availability, with each interview lasting approximately two hours. All interviews were audio-recorded and transcribed verbatim into a Microsoft Word document, then imported into the qualitative data analysis program ATLAS.ti version 22^26^ as a single hermeneutic unit to ensure that codes and categories corresponded to each other^27^.

The transcripts were read thoroughly, selecting quotations corresponding to the planned thematic areas. They were coded and grouped into categories based on thematic similarity.

A phenomenological analysis^28^ was conducted in three phases:

a. Description: In this phase, both participants and their statements were presented and described in general terms. For this purpose, the framework proposed by Patton^29^ was applied, which encompasses the following categories: experiences, behaviors, opinions, values, feelings, sensations, and knowledge.

b. Reduction: In this phase, the constituent elements of basic lifeworld existentials and emergent categories were coded and categorized.

c. Interpretation: In this phase, the meaning and significance of the experience of being a worker with a disability were constructed.

To ensure the credibility, coherence, and accuracy of the results, the trustworthiness criteria established by Prado et al.^30^ and Guba^31^ were applied:

1. Credibility: Each interview was rigorously and accurately transcribed following a phenomenological-constructivist perspective that enabled a deep, contextualized understanding of participants’ experiences. Immersion in the phenomenological context allowed the identification of nuances and meanings attributed by WWDs to their workplace experiences.

2. Transferability: A comprehensive description of the inclusion and exclusion criteria, as well as the recruitment methodologies, was provided to allow other researchers to assess the applicability and relevance of the results in comparable settings. This level of precision ensures that the specificities of the context and sample are clearly comprehensible and replicable in future studies.

3. Dependability: The sample was selected rigorously and transparently. The data analysis followed a structured phenomenological framework encompassing the phases of description, reduction, and interpretation. This ensured a systematic and consistent analysis of the experiences described by the participants.

4. Confirmability: Confidentiality was guaranteed, maintaining participants’ anonymity throughout the study, ensuring the impartiality of the results. Furthermore, three researchers reviewed and validated the data and analyses to maintain objectivity. Likewise, some participants verified the results, confirming that the findings accurately represented their experiences, which added an additional level of validity to the identified themes and subthemes.

All data collected is available for free access and consultation at Mendeley Data^32^.

**Ethical considerations**

All participants were informed about the objectives of the research and the data collection technique. It was explicitly stated that participation was voluntary, and both verbal and written informed consent with the participants’ signature were requested. The anonymity of both participants and companies that employed them was safeguarded. This research complied with the ethical principles for research involving human participants outlined in the Declaration of Helsinki and in Resolution 8430 of 2003 issued by the Colombian Ministry of Health. The study was approved by the Ethics Committee of the Institución Universitaria Antonio José Camacho (approval code PI-0420).

## Results

A total of 49 quotations were identified, corresponding to 19 codes, which were grouped into four categories: worker adaptations (7), job characteristics (3), disability consequences at work (2), and worker aspects (7).

**Figure 1:**
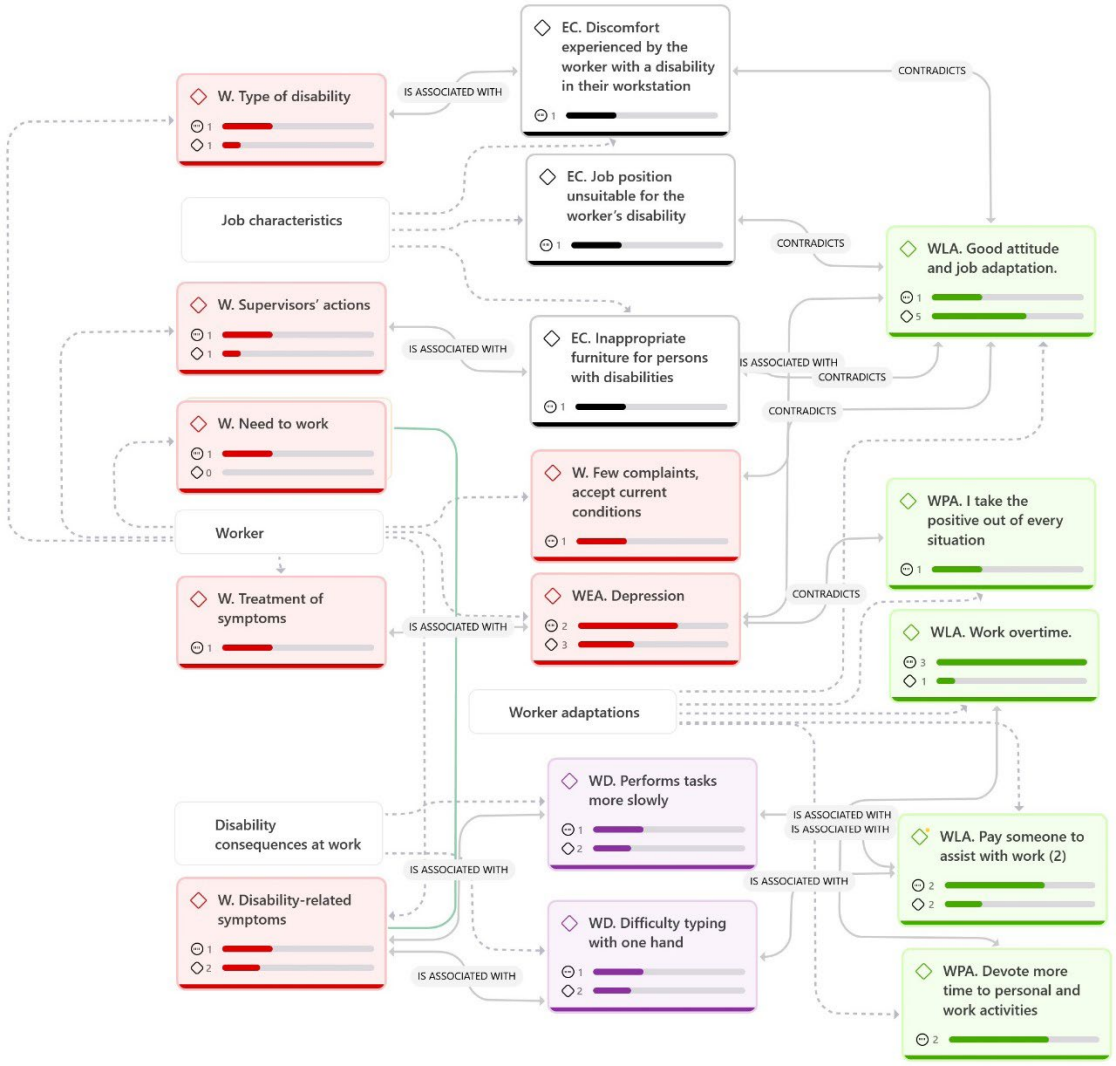
Analytical network of codes generated through qualitative data analysis using ATLAS. ti version 22.

As shown in Figure 1, the code “working overtime,” belonging to the category “worker adaptations,” was the most deeply rooted, followed by“devoting more time to personal and work activities,”“paying someone to assist with work,” and“depression.” In terms of code density, the codes“good attitude” and “adaptation to the job position” showed a stronger relationship with other codes analyzed.

*C: Ergonomic conditions, W: Worker, WEA: Emotional adaptations, WLA: Labor adaptations, WD: Worker difficulties, WPA: Worker positive adaptations.*

**Phase 1: Description**

Five workers with a disability, aged between 42 and 54, who were formally employed by a company, participated in the study. The group comprised two women and three men. Four participants had motor disabilities, and one had a visual impairment. Regarding educational background, two said they had university studies, two were technicians, and one had high school education. They declared that they had been employed by the company for more than 5 years. Four of them acquired their disabilities after being hired, except for the worker with visual impairment. Most participants were employed under one-year fixed-term contracts. The participants were receptive and willing to talk about their experiences. They used colloquial terms and popular slang (see Table 2).

**Table 2:** Frequency of complications

Age	Sex	Type of disability	Disability	Education	Type of employment contract	Length of service in the position	Years with disability
42	M	Sensory	Visual impairment	University	Open-ended	5 years	32 years
51	F	Motor	Degenerative arthritis	Secondary	1-year fixed-term	8 years	7 years
46	M	Motor	Brachial plexus injury with paralysis	Technical	1-year fixed-term	12 years	10 years
54	M	Motor	Right-hand amputation	University	1-year fixed-term	6 years	4 years
48	F	Motor	Right-hand amputation	Technical	1-year fixed-term	5 years	4 years

**Second phase: Reduction**

**Lived space:** Workers reported that, although they are independent in their mobility, they appreciate support from others:

*“…my wife drives me, but it’s not out of necessity. I can get around on my own or with someone else. I’m independent, but if someone makes things easier for me, I don’t say no.” (TD3).*

At the same time, they expressed that requests for workplace adaptations or accommodations have often gone unaddressed:

*“...in that sense, in the work environment, the necessary adaptations haven’t been made. I don't know why that is.” Contrary, “there are even more obstacles now. We used to have a spacious, well-organized office. Now, due to company's needs, more people have been hired, more desks have been added, and filing cabinets have been moved around. All of that has become an obstacle to getting around.” (TD1)*

Overall, participants noted that the physical layout of their workplaces is not designed to facilitate the mobility of WWDs. Instead, it tends to prioritize design, adaptation of space to particular needs of the company, and even clutter and disorganization:

“If you ever needed to rush out, it wouldn’t be possible, and sometimes staff leave plant pots, boards, or boxes in the way, and you end up bumping into them. Also, there are these decorative columns at the company entrance— very nice to look at, but for people with visual impairments, they just make orientation harder.” (TD1)

*Workers emphasized maintaining a positive attitude and striving to adapt to their jobs: “…well, for me, it hasn't been difficult at all. You know that any disability comes with certain limitations.” (TD1)*

**Lived body**

The body is experienced through the care required to prevent injuries, accidents, or further deterioration of health:

*“I have to be careful, because sometimes I can’t feel my whole arm. I could hurt myself or bump into something without meaning to.” (CTD1).*

Workers reported that certain tasks are more difficult to perform due to their disabilities, and how this affects them emotionally:

*“…No doubt, operating a keyboard with one hand is tedious, but I do it without complaining.” (TD2)*

*“Your self-esteem and motivation take a hit when this kind of injury happens. The moment you realize you can’t move one of your limbs anymore, you already start to feel bad.” (TD2)*

**Lived human relations**

When it comes to lived human relations at work, employees emphasize that they are often viewed with admiration by their colleagues:

*“...in terms of work, things are great, because there’s a respectful atmosphere, and people admire what we’ve accomplished and how we carry ourselves.” (CDT2)*

Overall, workers expressed that both colleagues and supervisors are willing to provide support. However, they also tend to avoid imposing on others when they need help:

*“…the friendship and camaraderie in the office—we try to help each other out (…) I have an excellent relationship with my boss, and the trust people place in you at work ensures a good relationship and helps you do your job in the best possible way.” (TD1)*

**Lived time**

Workers acknowledged that, because of their limitations, certain tasks, both work-related and personal, require more time to complete:

*“…lifting heavy objects, typing quickly on the computer keyboard, doing two things at once, typing quickly on my phone, or organizing my personal and work items—these are things I do, but they take me longer than normal.” (TD2)*

**Working conditions**

Regarding fairness, compensation, and opportunity, one worker noted:

*“Another activity I’m involved in is leading the union organization. I take part in meetings and social activities with the workers.” (TD1)*

In one of the companies, a specialized adaptation was made to meet the workers' needs to facilitate their inclusion:

*“…a reading system and a scanner were installed (…) they give me support whenever I need it.” (CTD2).*

Companies have the same selection criteria for all employees, regardless of disability status, and offer equivalent contractual conditions, which may be adjusted in some cases according to job performance:

*"...although some people have received a little introduction, like, ‘people with certain visual impairments should be treated this way,’ because the only difference is that if I go somewhere, they might just say, ‘sit down,’ without telling me where the chair is— ‘to your left,’ ‘to your right,’ or ‘behind you.’ Or they don’t think to guide me by placing my hand on the chair’s back so I can locate it. These are techniques most people don’t know or understand. That is why, with the people I trust, when they are with me, I have to explain these things to them in order to make it easier for them to get along. Explain that they should guide us, tell us, ‘Hey, we're here! in such-and-such a place’ or ‘there’s an obstacle here.’” (TD1).*

Among the unfavorable conditions, workers mentioned a lack of follow-up and evaluation in the reintegration and mandatory adaptation. Workplace accommodations are often postponed until office renovations, which can take years:

*“...no, well, what I'm telling you is that now that the office is going to be remodeled, they’re finally thinking about the workspace—but how long have they been talking about that?” (TD3).*

Workers commented that, although attempts are made to ensure that all workers have the same rights and opportunities, it remains more difficult for them:

*“…getting a promotion is too complicated. There have been cases—I’m an example of that. I started from the bottom. I was a receptionist, and I got promoted, but opportunities like that don’t come up often.” (TD1)*

WWDs viewed adaptation as a process that they must undertake individually, rather than as a set of actions and conditions that the company and its employees must facilitate:

*“...adaptations should be made with everyone in mind, but I’m the only one, they’re not going to think about just one person, so I have to adapt. It’s a company with very collaborative and supportive employees, but there’s no real intention to make specific adaptations because it’s very small and doesn’t own its facilities. So, they say it’s not worth making changes because it would be practically investing in a place that doesn’t belong to them.” (TD1).*

**Interpretation**

The working experience of PWDs in the formal sector is constructed as a process of daily adaptation, shaped by material, symbolic, and social conditions that result in limited inclusion. Based on Alfred Schutz's social phenomenology, this experience is interpreted as the result of the interaction between “because motives” (past experiences of exclusion or marginalization) and “in-order-to motives” (the pursuit of independence, economic stability, and self-fulfillment) that guide the actions of WWDs in the world of work. This meaning structure leads them to accept standard working conditions, even when these do not provide genuine accommodations, prioritizing personal commitment over organizational barriers.

In **lived space**, participants’ narratives reveal inaccessible workplaces, where design decisions privilege aesthetics or general functionality over the mobility of diverse bodies. Overcrowded offices, spatial disorder, and the absence of specific accommodations reinforce a sense of structural exclusion. Nevertheless, WWDs appreciate any assistance offered, even when they do not formally request it, keeping the logic of self-management.

The **lived body** emerges as a vulnerable yet resilient dimension that demands continuous care. Workers adopt preventive practices to avoid aggravating their conditions and modify their work methods to align with their physical capacities, even when this entails pain, discomfort, or emotional strain. Self-esteem and motivation, deeply affected by disability, are reconstructed through individual effort and the drive to sustain performance.

**Lived time** is redefined as everyday activities that were once simple now require more time and concentration. This prolonged temporality, however, does not result in institutional adjustments; rather, it is self-managed by workers who endeavor to meet the same standards as their non-disabled peers, frequently at the expense of their own well-being.

Within **lived human relations**, perceptions of respect and admiration from colleagues and supervisors coexist with a lack of practical knowledge about how to interact with PWDs. The absence of induction or training generates subtle forms of dependency, which WWDs strive to minimize so as not to “bother” others, thereby reinforcing relationships marked by asymmetric intersubjectivity.

**Working conditions** reflect a form of formal equality—same contracts, selection criteria, and requirements—that does not necessarily translate into actual equity. Opportunities for promotion and advancement remain mediated by invisible barriers, such as perceived risk, lack of reasonable accommodations, and the notion that investing in adaptations for a single individual is “not worthwhile.” The statement “conditions are the same, nothing special” sums up this contradiction: adaptation is understood as a personal duty rather than an organizational responsibility.

In summary, the experience of WWDs is not limited to performing tasks under adverse conditions; it represents a way of meaning-making and belonging within an environment that demands constant adaptation. This form of inclusion, which is more symbolic than structural, requires reexamining not only physical and contractual conditions but also the collective imaginaries that shape how disability is perceived, valued, and addressed in the workplace.

## Discussion

For persons with disabilities (PWDs), the experience of being a formally employed worker stems from the opportunity to remain connected, independent, and valuable to society^2^,^4^. Companies that have incorporated WWDs into their workforce acknowledge their potential and have provided incentives, fair treatment, and compensation in most areas of employment. However, that is less true in areas of participation, which often require investments that are unfeasible for companies from a cost-benefit analysis perspective, considering that, in relation to the total number of workers, very few benefit from them^1^,^5^.

There was a perceived need to strengthen inclusion processes through educational initiatives aimed at coworkers, focusing on how to interact with and support workers with different types of disabilities. Workers undergo mandatory and individual adaptation processes to remain in their job positions. They tend to accept and adapt to given job conditions, perceiving reasonable accommodations as desirable; however, they understand that companies generally do not have these resources available, so it is up to the workers to adapt^4^,^5^.

Despite the adverse ergonomic conditions of the workplace, there is a strong sense of belonging and gratitude toward their company, colleagues, and bosses, accompanied by mutual recognition of responsibility and appreciation for the effort invested in performing tasks despite limitations^4^,^5^,^7^,^33^. Although the workplace inclusion processes described in this article were successful for both employers and WWDs, they were developed empirically and intuitively. None of the participating companies had an established procedure to address the specificities of this type of hiring.

Integration appears as an alternative to adaptation of the workplace for employees with disabilities. However, once again, the argument was that the good performance and interaction of WWDs result from certain factors related to the receptiveness among those in the workplace, rather than from modifications or physical accommodations. WWDs tend to generate high expectations and satisfaction among all parties involved in the process. It is necessary to train entire work teams that interact with employees with disabilities and to promote more flexible attitudes to achieve successful inclusion^34^. As one participant summarized, “Attitude is key to the perception of disability and to performance in the workplace.”

The findings of this research, analyzed through the lens of Schutz’s social phenomenology, reveal how WWDs construct their working life-world (Lebenswelt) through everyday experiences of self- adaptation^35^. The absence of reasonable adaptations in their work environments has led these workers to develop a distinctive“stock of knowledge”^36^ expressed through individualized adaptation strategies that allow them to navigate their workspaces. Intersubjectivity, a central concept in Schutz's theory, is evident in the way participants create shared meanings about their work experience, captured in the statement “The conditions are the same, nothing special,” which suggests a typification of their work reality where adaptation is perceived as a personal duty rather than an organizational responsibility^37^.

This perspective aligns with Morujão’s^38^ notion of mental constructs that people use to make sense of their social experiences. The results also reflect how WWDs have developed what Tisott et al.^39^ refer to as mutual understanding within their work environments; however, this understanding is built primarily through their own adaptation efforts rather than institutional adjustments. This dynamic reveals a tension between the need for reasonable accommodations and the actual expectation that WWDs conform to preexisting environments, highlighting the challenge of creating genuinely inclusive workplaces.

Finally, this study has some limitations that should be acknowledged. First, work experiences were not explored separately depending on whether the disability was acquired before or after joining the company. This distinction could significantly influence inclusion processes and shed light on understanding the phenomenon, since the duration of the disability and its origin could be an important differentiating factor in the company's response to the illness. Second, the sample consisted exclusively of individuals with motor and visual impairments, which limits the transferability of the findings to other types of disability.

## Conclusions

WWDs adapt to their working conditions regardless of whether they have access to an inclusive environment or not. They perceive this process as an individual commitment in response to the opportunity to work, rather than as a set of actions and conditions that companies and co-workers must enable. Once integrated into the workplace, they perform their duties without functional, productive, or contractual differences compared to their peers without disabilities.

Future research could delve deeper into comparing different career trajectories based on when the disability was acquired, as well as exploring other sensory, intellectual, or psychosocial conditions. It is also recommended to advance toward studies encompassing greater organizational and sectoral diversity, which would provide insight into how inclusion varies depending on the type of enterprise or organizational culture. It is also necessary to investigate the experiences of teams that support WWDs and move toward longitudinal designs that make it possible to observe the evolution of inclusion processes over time.
